# 6-sulfo sialyl Lewis X is the common receptor determinant recognized by H5, H6, H7 and H9 influenza viruses of terrestrial poultry

**DOI:** 10.1186/1743-422X-5-85

**Published:** 2008-07-24

**Authors:** Alexandra S Gambaryan, Alexander B Tuzikov, Galina V Pazynina, Julia A Desheva, Nicolai V Bovin, Mikhail N Matrosovich, Alexander I Klimov

**Affiliations:** 1 Chumakov Institute of Poliomyelitis and Viral Encephalitides, RAMS, 142782 Moscow, Russia; 2 Shemyakin Institute of Bio-organic Chemistry, RAS, 117997 Moscow, Russia; 3 Institute of Experimental Medicine, RAMS, 197376 St. Petersburg, Russia; 4 Institute of Virology, Philipps University, 35043 Marburg, Germany; 5 Influenza Division, Centers for Disease Control and Prevention, Atlanta, GA 30333, USA

## Abstract

**Background:**

Influenza A viruses of domestic birds originate from the natural reservoir in aquatic birds as a result of interspecies transmission and adaptation to new host species. We previously noticed that influenza viruses isolated from distinct orders of aquatic and terrestrial birds may differ in their fine receptor-binding specificity by recognizing the structure of the inner parts of Neu5Acα2-3Gal-terminated sialyloligosaccharide receptors. To further characterize these differences, we studied receptor-binding properties of a large panel of influenza A viruses from wild aquatic birds, poultry, pigs and horses.

**Results:**

Using a competitive solid-phase binding assay, we determined viral binding to polymeric conjugates of sialyloligosaccharides differing by the type of Neu5Acα-Gal linkage and by the structure of the more distant parts of the oligosaccharide chain. Influenza viruses isolated from terrestrial poultry differed from duck viruses by an enhanced binding to sulfated and/or fucosylated Neu5Acα2-3Gal-containing sialyloligosaccharides. Most of the poultry viruses tested shared a high binding affinity for the 6-sulfo sialyl Lewis X (Su-SLe^x^). Efficient binding of poultry viruses to Su-SLe^x ^was often accompanied by their ability to bind to Neu5Acα2-6Gal-terminated (human-type) receptors. Such a dual receptor-binding specificity was demonstrated for the North American and Eurasian H7 viruses, H9N2 Eurasian poultry viruses, and H1, H3 and H9 avian-like virus isolates from pigs.

**Conclusion:**

Influenza viruses of terrestrial poultry differ from ancestral duck viruses by enhanced binding to sulfated and/or fucosylated Neu5Acα2-3Gal-terminated receptors and, occasionally, by the ability to bind to Neu5Acα2-6Gal-terminated (human-type) receptors. These findings suggest that the adaptation to receptors in poultry can enhance the potential of an avian virus for avian-to-human transmission and pandemic spread.

## Background

The recent pandemic threat caused by the widespread circulation of H5N1 avian influenza viruses and their occasional transmission to humans as well as human infections caused by chicken H9N2, H7N7 and H7N3 viruses highlighted the need for a detailed study of host restriction mechanisms of influenza viruses. Numerous studies support the concept that alteration of the receptor specificity of an avian virus is essential for its transmission into humans as well as for human-to-human transmission and pandemic spread (reviewed in ref. [1,2]).

The history of research into the receptor binding phenotypes of influenza viruses can be divided into two periods: before and after 1997 when first human infections with chicken H5N1 viruses were documented. Before 1997, it was established that human influenza viruses recognize Neu5Acα2-6Gal-terminated receptors, avian viruses recognize Neu5Acα2-3Gal-terminated receptors while swine viruses recognize both of them [3-8]. It was shown that the receptor-binding site (RBS) of the hemagglutinin (HA) of avian viruses is evolutionally very stable. In addition to eight amino acids forming the HA RBS, which are conserved in all influenza A viruses (positions 97, 98, 134, 139, 153, 183, 184 and 195; H3 numbering is used here and throughout the paper), there are six more amino acids conserved in HAs of duck viruses (positions 138, 190, 194, 225, 226 and 228), and these are positions where human HAs are different from duck viruses [7]. Virus receptor binding specificity was found to correlate with the level of expression of relevant sialic acids determinants on the target cells of different host species. Thus, epithelial cells of human airway epithelium were shown to express high amounts of Neu5Acα2-6Gal-terminated sialyloligosaccharides, duck intestinal epithelium predominantly contains Neu5Acα2-3Gal-terminated receptors while swine tracheal epithelium contains both receptor types [8,9]. It was hypothesized that alteration of receptor specificity of avian viruses in some intermediate host, such as swine, might facilitate their transmission to humans [10].

After 1997, it became clear that avian H5N1 viruses are capable of replicating in humans [11,12] despite their avian-virus-like preference for Neu5Acα2-3Gal-containing receptors and lack of binding to human-type receptors [13]. It was shown afterwards that human airway epithelial cells express 2-3-linked sialic acid receptors with a density sufficient for the entry and replication of avian viruses [14,15].

Furthermore, a Eurasian lineage of poultry H9N2 viruses was discovered, which recognized Neu5Acα2-6Gal-terminated sialyloligosaccharides, thus indicating that some avian influenza viruses may display a human-virus-like receptor specificity [16-18]. It was also demonstrated that chicken and quail intestinal cells contain both Neu5Acα2-3Gal and Neu5Acα2-6Gal sialyloligosaccharides, in contrast to duck cells that contain only Neu5Acα2-3Gal [19-23].

Although the Neu5Acα2-3Gal receptor specificity is shared by the majority of avian viruses, viruses adapted to different avian species can differ in their ability to recognize the third saccharide and more distant moieties of Neu5Acα2-3Gal-terminated receptors. For example, duck viruses of various subtypes preferentially bound to glycoprotein O-chain trisaccharide Neu5Acα2-3Galβ1-3GalNAcα, whereas H5N1 chicken viruses preferred receptors with inner β-N-acetylglucosamine moiety, Neu5Acα2-3Galβ1-4GlcNAcβ [20]. Sulfation of the saccharide core produced no effect on binding of duck viruses, whereas chicken and human viruses isolated in 1997 in Hong Kong demonstrated an extraordinarily high affinity for sulfated trisaccharide Neu5Acα2-3Galβ1-4(6-HSO_3_)GlcNAc (Su-3'SLN) [24,25].

In the present study, we characterized the receptor-binding specificity of a broad set of influenza A viruses from wild aquatic birds, poultry, pigs and horses.

## Results

### Receptor-binding specificity

**Figure 1:**
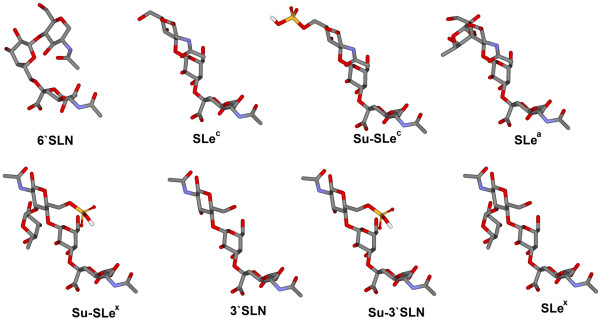
**Molecular models of sialyloligosaccharides**. The models depict sialyloligosaccharide parts of glycopolymers that were tested for their binding to influenza viruses. Corresponding structural formulas are given in the Table 1. The figures were generated using Discovery Studio ViewerPro5.0 software (Accelrys Inc.).

**Figure 2:**
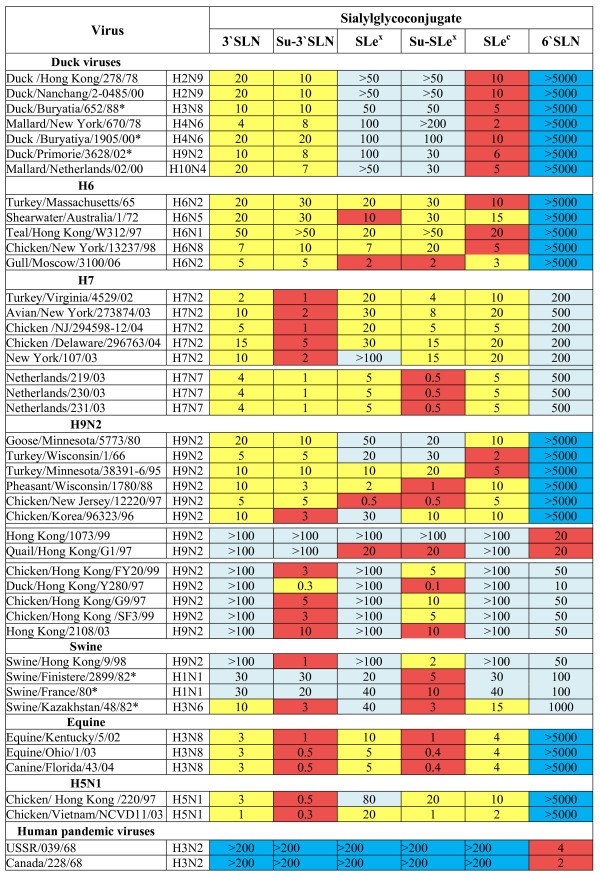
**Binding affinity constants of virus complexes with sialylglycopolymers**. The constants were determined as described in the Methods and were expressed in μM of sialic acid. Higher values of constants correspond to lower binding affinities. The data were averaged from 3 sets of experiments. Standard errors did not exceed 50% of the mean values. Viruses labelled with asterisk were kindly provided by Dr. S. Yamnikova, the Ivanovsky Institute of Virology, Moscow, Russia. Colours depict relative levels of binding for each individual virus: red – maximal binding; yellow – good binding; pale cyan – weak binding; blue – no detectable binding.

To determine the receptor-binding specificity of avian and mammalian influenza viruses, we tested their binding to 9 distinct polymeric glycoconjugates (see Fig. 1 and Table 1 for structural formulas and abbreviations). One of the glycoconjugates harboured 6-linked sialyloligosaccharide, Neu5Acα2-6Galβ1-4GlcNAc (6'SLN). The oligosaccharide parts of the other glycopolymers shared the same terminal Neu5Acα2-3Gal moiety but differed: (i) by the type of the bond between galactose and the next sugar residue (β1–3 or β1–4), (ii) by the nature of this residue (GlcNAcβ or GalNAcα), and (iii) by constituents at different positions on the GlcNAc ring (fucose or/and sulfo group). All studied oligosaccharide structures have been found in natural glycoproteins or glycolipids [26]. Virus binding to glycoconjugates was determined in a competitive solid-phase assay and expressed in terms of binding affinity constants (Fig. 2).

**Table 1:** Structure of sialyloligosaccharide parts of glycopolymers

Sialyloligosaccharide	Abbreviation
Neu5Acα2-3Galβ1-4GlcNAcβ	3'SLN
Neu5Acα2-3Galβ1-4(6-HSO_3_)GlcNAcβ	Su-3'SLN
Neu5Acα2-3Galβ1-4(Fucα1-3)GlcNAcβ	SLe^x^
Neu5Acα2-3Galβ1-4(Fucα1-3)-(6-HSO_3_)GlcNAcβ	Su-SLe^x^
Neu5Acα2-3Galβ1-3GlcNAcβ	SLe^c^
Neu5Acα2-3Galβ1-3(6-HSO_3_)GlcNAcβ	Su-SLe^c^
Neu5Acα2-3Galβ1-3GalNAcα	STF
Neu5Acα2-3Galβ1-3(Fucα1-4)GlcNAcβ	SLe^a^
Neu5Acα2-6Galβ1-4GlcNAc	6'SLN

Each of the tested viruses bound SLe^c^, Su-SLe^c ^and STF with the same affinity and none of the viruses discriminated between SLe^x ^and SLe^a^. We, therefore, do not show here the binding data for Su-SLe^c^, STF and SLe^a^. The patterns of viral binding to the panel of receptor analogues varied significantly among viruses of different subtypes and host species (Fig. 2), however, several distinctive groups of viruses with typical receptor binding phenotypes could be recognized as described below.

#### Viruses of various subtypes isolated from wild ducks

These viruses displayed the highest binding affinity for glycoconjugates with β(1–3) linkage between Neu5Acα2-3Gal disaccharide fragment and the next GlcNAc residue, i.e., SLe^c^, Su-SLe^c ^and STF. Other characteristic features of duck viruses were their low affinity for fucosylated sialyloligosaccharides SLe^a ^and SLe^x^, nearly equal affinity for sulfated and non-sulfated sialyloligosaccharides, and a lack of appreciable binding to 6'SLN.

#### Viruses with H6 HA

Five viruses with H6 HA tested in this study were isolated from different avian species (turkey, shearwater, teal, chicken and gull). Unlike typical duck viruses, all H6 viral isolates efficiently bound to fucosylated sialyloligosaccharides SLe^x ^and SLe^a^.

#### Viruses with H7 HA

Viruses of H7 subtype from two evolutionary lineages were tested: 1) American avian H7N2 viruses and closely related human isolate A/New York/107/03 (H7N2) [27], and 2) Eurasian H7N7 human isolates that were transmitted to humans from infected poultry during the 2003 outbreak in the Netherlands [28]. Viruses from both lineages showed enhanced binding to sulfated sialyloligosaccharides with the Galβ1-4GlcNAcβ core. The American viruses displayed the highest affinity for Su-3'SLN, whereas the H7N7 viruses from the Netherlands had particularly high affinity for Su-SLe^x^. It was found unexpectedly that all H7 viruses tested displayed moderate binding affinity for human-type receptor 6'SLN (Fig. 2).

#### H9N2 viruses

The H9N2 viruses tested could be arbitrarily separated into three groups, North American viruses and distantly related virus A/Chicken/Korea/96323/96 [29] and two evolutionary lineages of poultry viruses from Southeast Asia, G1 and G9 [16-18].

Receptor-binding affinity of A/Goose/Minnesota/5773/80 and A/Turkey/Wisconsin/1/66 was similar to that of A/Duck/Primorie/3628/02 (H9N2) and duck viruses of other subtypes: they preferentially bound to SLe^c ^and bound poorly to fucosylated sialyloligosaccharides. Other North American viruses such as A/Chicken/New Jersey/12220/97 and A/Pheasant/Wisconsin/1780/88 showed high binding affinity for SLe^a^, SLe^x ^and Su-SLe^x^. A/Chicken/Korea/96323/96 had an increased affinity for sulfated sialyloligosaccharides Su-3'SLN and Su-SLe^x^. None of these viruses bound 6'SLN.

In contrast to North American viruses, Asian isolates from G1- and G9- lineages bound to 6'SLN. The binding pattern of the human isolate A/Hong Kong/1073/99 (H9N2) resembled that of pandemic human viruses A/USSR/039/68 and A/Canada/228/68 (see Fig. 2, bottom lines): all these three viruses strongly bound to 6'SLN and did not appreciably bind to Neu5Acα2-3Gal-containing oligosaccharides. A/Quail/Hong Kong/G1/97 demonstrated high affinity for 6'SLN, SLe^x ^and Su-SLe^x ^and did not bind to any of non-fucosylated Neu5Acα2-3Gal-containing receptors. All other Asian poultry viruses tested displayed moderate binding to 6'SLN, bound much stronger to sulfated receptors Su-3'SLN and Su-SLe^x ^and did not bind at all to 3'SLN, SLe^c^, SLe^x^, and Su-SLe^c^.

#### Swine viruses

Four viruses isolated from pigs were tested. A/Swine/Hong Kong/9/98 belonged to the G9 clade of H9N2 viruses, A/Swine/Finistere/2899/82 and A/Swine/France/80 represented the European avian-like swine virus lineage and A/Swine/Kazakhstan/48/82 was a sporadic avian-like H3N6 isolate. A common feature of these viruses was their high affinity for Su-SLe^x ^and a moderate affinity for 6'SLN.

#### Equine viruses

Equine H3N8 viruses including the equine-like canine isolate A/Canine/Florida/43/2004 [30] showed a strong binding affinity for Neu5Acα2-3Gal receptors, preferring sulfated ones, Su-3'SLN or Su-SLe^x^.

#### H5N1 Asian viruses

Viruses of this group were extensively analyzed in our previous studies [24,25]. Two typical chicken isolates were tested here for a comparison with other poultry viruses. Both A/Chicken/Hong Kong/220/97 and A/Chicken/Vietnam/NCVD11/03 revealed increased affinity for Su-3'SLN. The latter virus in addition showed a high affinity for Su-SLe^x^.

### Analysis of HA amino acid sequences and molecular modelling of the complexes of Su-SLe^x ^with H3, H7 and H9 HA

**Figure 3:**
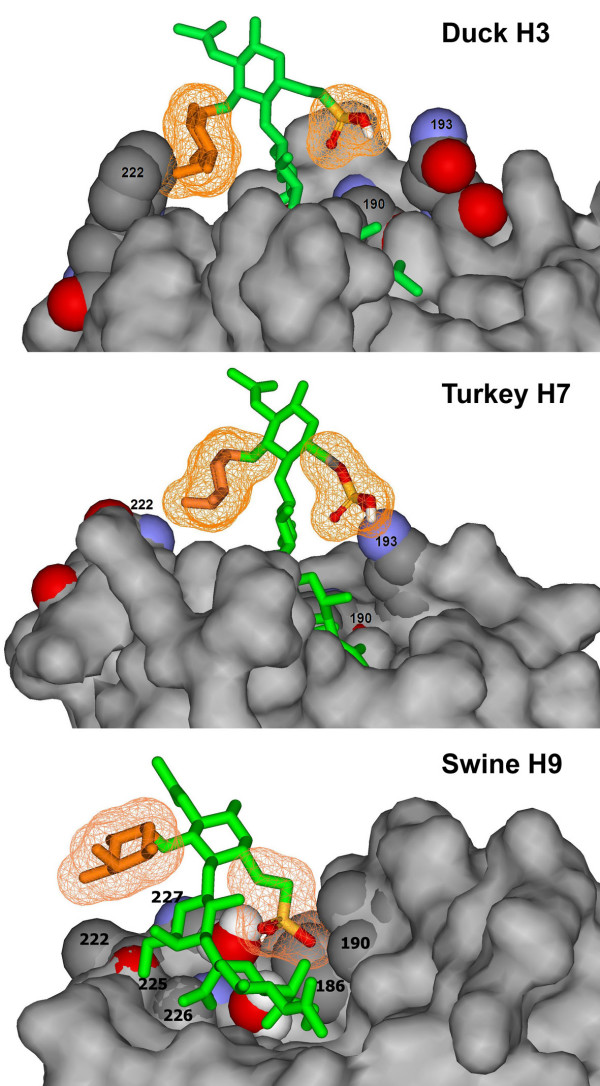
**Models of complexes of H3, H7 and H9 hemagglutinins with Su-SLe^x^**. The models were generated as described in the Methods using the crystal structures of the HAs of the viruses A/Duck/Ukraine/1/63 (H3N8) [32], A/Turkey/Italy/02 (H7N1) [33] and A/Swine/Hong Kong/9/98 (H9N2) [37]. The fucose moiety and the sulfo group of Su-SLe^x ^are shown as mesh surfaces. Amino acid residues described in the text are numbered.

The characteristic feature of the duck viruses tested herein was their poor binding to fucosylated sialyloligosaccharides (Fig. 2, upper part). This receptor-binding phenotype agreed with that described earlier for a variety of viruses from wild ducks [20,24,31]. In order to understand the molecular basis of this phenotype, we modelled a putative disposition of the fucosylated receptor Su-SLe^x ^in the receptor-binding site of the HA of Duck/Ukraine/1/63 (H3N8) [32]. The modelling predicted that the fucose moiety would come into a significant sterical conflict with the side chain of Trp222 (Fig 3). We next compared the HA sequences of more than 400 duck influenza viruses of H1, H2, H3, H4, H5, H8, H9, H10, H11 and H14 subtypes available from the Genbank. All of these viruses had a bulky amino acids (Arg, Lys, Trp, Leu, or Gln) in position 222 of the HA. We suggest on this basis that partial overlap of the fucose moiety with the bulky amino acid in position 222 could be a universal mechanism that reduces the capability of duck viruses to bind fucosylated receptors.

**Figure 4:**
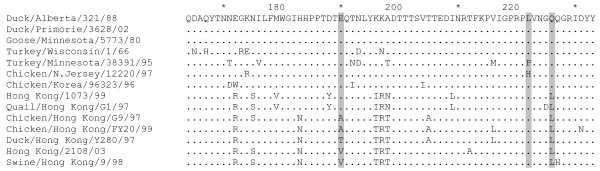
**Partial HA amino acid sequences of H9N2 viruses**. The sequences were obtained from GenBank. Differences with respect to the top sequence are shown. Amino acids in positions 190, 222, and 226 are highlighted. The figure was generated with GeneDoc 2.6 software [50].

Our analysis of 68 published H6 HA sequences revealed that 67 of them have Ala222. This finding suggests that a relatively good binding of H6 viruses to fucosylated sialyloligosaccharides SLe^x ^and SLe^a ^(Fig. 2) could be explained by a lack of interference between the fucose moiety and the short side chain of the alanine in position 222 of the HA. Essential role of amino acid in position 222 in the binding of fucosylated receptors was also supported by the comparison of HA sequences of the H9N2 viruses, A/Goose/Minnesota/5773/80 and A/Chicken/New Jersey/12220/97 (Fig. 2 and Fig. 4). The latter virus had His222 and bound SLe^x ^100-times better than the former virus (Leu222).

The high binding affinity of H7 viruses to sulfated sialyloligosaccharides suggested that the sulfo group interacts with some charged amino acid residue in the receptor-binding site. To test this possibility, we modelled potential contacts of Su-SLe^x ^with the receptor-binding pocket of avian H3 and H7 HAs [32,33]. In the case of H3 duck virus, the sulfo group faced towards solution and did not form obvious direct contacts with the protein. However, in the case of the H7 HA, the sulfo group of Su-SLe^x ^was located in a close proximity to the side chain of Lys193, which is highly conserved among viruses with H7 HA (Fig 3). This finding suggests that enhanced affinity of H7 viruses for Su-3'SLN and Su-SLe^x ^is due to favourable charged interactions between the sulfo group of the receptor and amino group of Lys193. The same mechanism is likely responsible for the high affinity for Su-3'SLN of H5N1 (Gambaryan et al., 2004, 2006) and H3N8 equine viruses (Fig. 2) since viruses of both these groups have lysine in position 193.

South-eastern Asian H9N2 viruses have multiple amino acid substitutions in the receptor-binding region of the HA, most notably, the mutation Gln226Leu [34] (Fig. 4). The typical human-virus-like receptor specificity of A/Hong Kong/1073/99 (H9N2) is in a good agreement with the notion that a single Gln226Leu replacement shifts the receptor specificity from recognition of Neu5Acα2-3Gal to recognition of Neu5Acα2-6Gal receptors [35,36]. A/Quail/Hong Kong/G1/97 (H9N2) differs from A/Hong Kong/1073/99 (H9N2) by the Gly225Asp substitution (Fig. 4) that markedly enhances the affinity of this virus for fucosylated 3-linked receptors SLe^x ^and Su-SLe^x ^and leads to a rather an unusual receptor-binding phenotype (Fig. 2).

The H9N2 viruses of G9-lineage harbour substitutions Gln226Leu and Glu190Ala/Thr/Val in the HA [18,34] (Fig. 4). Viruses with Ala or Thr in position 190 bound Su-3'SLN and Su-SLe^x ^with the highest affinity and demonstrated moderate affinity for 6'SLN (Fig. 2). As these viruses did not noticeably bind to Su-SLe^c^, specific orientation of the sulfo group rather than its negative charge alone seems to be essential for the binding. We used the crystal structure of the HA of A/Swine/Hong Kong/9/98 (H9N2) (G9-lineage) in complex with 3-linked receptor [37] for the modelling of H9 HA interactions with Su-SLe^x ^(Fig. 3). Due to the amino acid substitutions in positions 226 and 190 of the H9 HA, the conformation of 3-linked galactose in this complex differs from that in the H3 avian HA [32,37], leading to corresponding differences in the putative disposition of Su-SLe^x ^(compare H3 and H9 complexes in Fig. 3). In the H9 HA, the fucose moiety shifts upwards resolving the steric interference with amino acid in position 222, whereas the sulfo group shifts downwards and fits into a cavity formed by amino acids in positions 190 and 186 and by the solvent water molecules bound to residues 98, 228 and 227 (PDB:1JSD[37]). This could explain why the substitutions Gln226Leu and Glu190Val in the H9 HA, that increased virus affinity for Neu5Acα2-6Gal, at the same time significantly enhanced its affinity for sulfated Neu5Acα2-3Gal-containing receptors, Su-3'SLN and Su-SLe^x^.

## Discussion

Although almost all avian viruses use the same terminal disaccharide Neu5Acα2-3Gal as receptor, the evolution of distinct virus lineages adapted to distinct avian species (wild ducks, gulls, or terrestrial poultry) has led to specialized abilities to recognize longer oligosaccharide chains. Thus, duck viruses have the highest affinity for SLe^c ^and STF (Neu5Acα2-3Galβ1-3GalNAcα). We demonstrated earlier that duck viruses bind strongly to gangliosides from duck intestine as well as to GD1a ganglioside, which is terminated by Neu5Acα2-3Galβ1-3GalNAcβ[7,19]. It is possible that gangliosides with this termination serve as functional receptors of influenza viruses in the duck intestine.

Our present study indicated that receptor specificity of viruses from different lineages adapted to quail and chicken differed from that of wild duck viruses. Sulfated and fucosylated 3'SLN is a suitable receptor for most of these poultry viruses. It was shown recently that bi-antennary a2-6/3 sialylated glycans with Galβ1-4GlcNAcβ core are major sialylated *N*-glycans expressed by intestinal epithelial tissues in both chicken and quail [23]. This fact is in accord with preferential binding of quail and chicken viruses to sialyloligosaccharides with Galβ1-4GlcNAcβ core.

Gull viruses appear to be adapted to fucosylated receptors, such as SLe^x ^[31]. We suggested earlier that the presence of glycine in HA position 222 of H13 and H16 viruses is essential for this binding phenotype [38]. In this study, we found that all tested H6 viruses, similarly to gull viruses, demonstrated enhanced affinity for SLe^a ^and SLe^x ^and that alanine in position 222 of the H6 HA is likely to play an essential role in this specificity. Since almost all sequenced H6 HA have Ala222, viruses of this subtype should be able to recognize receptor determinants that are optimal for duck (SLe^c^), gull (SLe^x^) and chicken (Su-SLe^x^) viruses. This feature of H6 viruses would agree with their known promiscuous host range [39]. Some American H9 poultry viruses with substitution in position 222 showed receptor binding phenotype that was similar to that of H13, H16 and H6 viruses. It could be speculated that mutations in position 222 that improve sterical accommodation of the fucose moiety could represent one general pathway for adaptation of duck viruses to fucosylated receptors present in gulls and chickens.

Asian H5N1 viruses, H7 poultry viruses and equine H3N8 viruses realized another pathway of adaptation to recognition of the Su-SLe^x ^determinant via the favourable electrostatic interactions between the sulfo group and the amino group of Lys193.

One more pathway of viral adaptation to Su-SLe^x ^can be achieved through a substitution of conserved glutamic acid in the HA position 190. Importantly, this substitution that leads to enhanced binding to Su-SLe^x ^is often accompanied by the enhanced viral binding to the human-type receptor 6'SLN. Thus, high affinity for Su-SLe^x ^and moderate affinity for 6'SLN was detected in this study for G9-like H9N2 viruses, and previously for H1N1 swine [40] and human [41] viruses. H7 viruses with high affinity for Su-SLe^x ^also showed detectable binding to 6'SLN (Fig. 2).

It is not clear whether the ability of H7 and H9 poultry viruses to bind to 6'SLN provides them with some evolutionary advantage. For example, the binding of these viruses to 6'SLN does correlate with the presence of 6'SLN-containing receptors in epithelial tissues of gallinaceous birds [19-23]. Alternatively, the ability of poultry viruses to bind to 6'SLN could be an accidental consequence of their adaptation for the binding to Su-SLe^x ^due to some sterical similarity between Su-SLe^x ^and 6'SLN in the regions of Neu5Ac-Gal glycosidic linkage and of the NAc-moiety of the GlcNAc residue (Fig. 1).

The binding data (Fig. 2) show that the receptor specificity of poultry H5, H7, and H9 viruses is similar to that of equine and avian-like swine viruses. If sulfated and fucosylated sialyloligosaccharides are present in the target cells of both terrestrial poultry and mammals, the adaptation of aquatic bird viruses to poultry could facilitate their replication in mammals, including humans.

## Conclusion

It is generally believed that alteration of the receptor specificity is a prerequisite for the highly effective replication and human-to-human transmission which characterize pandemic influenza viruses [1,2,42]. Here we found that several independent lineages of poultry influenza viruses differ from their precursors in aquatic birds by enhanced binding to 6-sulfo sialyl Lewis X and that this binding specificity is accompanied by the ability of the virus to bind to human-type receptor 6'SLN. We therefore suggest that the adaptation to Su-Sle^x ^receptor in terrestrial poultry could enhance the potential of an avian virus for avian-to-human transmission and pandemic spread.

## Methods

### Materials

Oligosaccharides conjugated with polyacrylamide (~30 kDa) were synthesized from spacered sialyloligosaccharides (spacer = -OCH2CH2CH2NH2 or -NHCOCH2NH2) and poly(4-nitrophenylacrylate) having m.w. 30 kDa by the method described earlier [43,44]. Spacered oligosaccharides were synthesized as described previously [45-47].

### Viruses

The majority of viruses in the study were from the repository of the Influenza Division, CDC, USA; some isolates were from the collection of the D.I. Ivanovsky Institute of Virology, Moscow. Viruses were grown in 9-day-old embryonated chicken eggs and were inactivated by treatment with beta-propiolactone as described previously [13]. The allantoic fluids were clarified by low-speed centrifugation; the viruses were pelleted by high-speed centrifugation, resuspended in 0.1 M NaCl, 0.02 M Tris buffer (pH 7.2) containing 50% glycerol, and stored at -20°C.

### The binding affinity of influenza viruses for sialylglycoconjugates

Receptor specificity of influenza viruses was evaluated in a competitive assay based on the inhibition of binding to solid-phase immobilized virus with bovine fetuin labelled with horseradish peroxidase [48]. The competitive reaction was performed at 2–4 oC for 30 min in PBS with 0.01% of Tween-20; 0.05% of BSA and 3 μM of the sialidase inhibitor 4-amino-Neu5Ac-en. The data were expressed in terms of affinity constants (K_aff_) formally equivalent to the dissociation constants of virus-receptor complexes. For the calculation of the constants, concentration of the sialic acid residues in the solution was used. Each set of experiments presented in the Fig. 2 was repeated three-four times with similar results. Data were averaged from 3 sets of experiments.

### Molecular models

Atomic coordinates of SLe^x ^(PDB:2KMB) [49], H7 HA (PDB:1TI8) [33], H9 HA (PDB:1JSD) [37] and H3 and H9 HA complexes with NeuAcα2-3Gal-containing pentasaccharide LSTa (PDB:1MQM and PDB:1JSH) [32,37] were obtained from Brookhaven Protein Data Bank. The molecular models were generated using DS ViewerPro 5.0 software (Accelrys Inc.).

The model of Su-SLe^x ^was constructed on the basis of SLe^x ^structure (PDB:2KMB), by replacing the hydrogen atom of the 6-OH group of GlcNAc by HSO_3 _group.

The models of Su-SLe^x ^in the receptor-binding sites of H3 and H9 HA were made by superimposing the galactose residue of the Su-SLe^x ^over the galactose residue of LSTa. The model of Su-SLe^x ^in the receptor-binding site of H7 HA was generated by superimposing the protein chain of the H3 HA complex with Su-SLe^x ^over the protein chain of H7 HA (PDB:1TI8). The OH groups of Tyr98, SG atoms of Cys139, CZ3 atoms of Trp153, CD atoms of Glu190 and CA atoms of Tyr 195 were used to align two proteins.

## Competing interests

The authors declare that they have no competing interests.

## Authors' contributions

Conception and design of the study, manuscript preparation (ASG, NVB, AIK, MNM); experimental work (ASG, ABT, GVP, JAD); co-ordination of the study (AIK, NVB).

## Disclaimer

The findings and conclusions in the report are those of the authors and do not necessarily represent the views of the funding agencies.
